# Clinical diagnostics and therapy monitoring in the congenital disorders of glycosylation

**DOI:** 10.1007/s10719-015-9639-x

**Published:** 2016-01-07

**Authors:** Monique Van Scherpenzeel, Esther Willems, Dirk J. Lefeber

**Affiliations:** Translational Metabolic Laboratory, Radboud University Medical Center, Geert Grooteplein 10, Nijmegen, 6525 GA The Netherlands; Department of Neurology, Radboud University Medical Center, Geert Grooteplein 10, Nijmegen, 6525 GA The Netherlands

**Keywords:** Congenital disorders of glycosylation, Glycomics, Protein-specific glycosylation, Transferrin

## Abstract

Abnormal protein glycosylation is observed in many common disorders like cancer, inflammation, Alzheimer’s disease and diabetes. However, the actual use of this information in clinical diagnostics is still very limited. Information is usually derived from analysis of total serum N-glycan profiling methods, whereas the current use of glycoprotein biomarkers in the clinical setting is commonly based on protein levels. It can be envisioned that combining protein levels and their glycan isoforms would increase specificity for early diagnosis and therapy monitoring. To establish diagnostic assays, based on the mass spectrometric analysis of protein-specific glycosylation abnormalities, still many technical improvements have to be made. In addition, clinical validation is equally important as well as an understanding of the genetic and environmental factors that determine the protein-specific glycosylation abnormalities. Important lessons can be learned from the group of monogenic disorders in the glycosylation pathway, the Congenital Disorders of Glycosylation (CDG). Now that more and more genetic defects are being unraveled, we start to learn how genetic factors influence glycomics profiles of individual and total serum proteins. Although only in its initial stages, such studies suggest the importance to establish diagnostic assays for protein-specific glycosylation profiling, and the need to look beyond the single glycoprotein diagnostic test. Here, we review progress in and lessons from genetic disease, and review the increasing opportunities of mass spectrometry to analyze protein glycosylation in the clinical diagnostic setting. Furthermore, we will discuss the possibilities to expand current CDG diagnostics and how this can be used to approach glycoprotein biomarkers for more common diseases.

**Contents**

1. Background......................................................................5
2. CDG diagnostics and gene identification........................7
3. Therapy development for CDG.......................................9
4. Novel glycoprotein biomarkers for CDG......................13
  4.1. The need for glycoprotein biomarkers beyond transferrin.............................................................13
  4.2. Potential glycoprotein biomarkers for diagnostic application in CDG............................................14
5. Mass spectrometry in glycoprotein analysis.................17
  5.1. Glycoprotein biomarker discovery.......................19
  5.2. Glycoprotein biomarker application in clinical diagnostics............................................................21
6. Outlook...........................................................................22
7. References......................................................................24

## Background

Protein glycosylation is a non-template driven, dynamic, and post-translational modification on top of protein synthesis. Glycans have important functions in many biological processes such as protein folding, clearance and cell-cell interactions. Considering glycosylation to be the final and top level after DNA and protein synthesis, glycosylation is the most informative level, being a product of the genome and environmental factors together. Therefore, the use of glycomics methods to read this information holds enormous potential for the development of unique biomarkers for human diseases. In several genetic and chronic diseases, like cancer [1–3], inflammation [4], Alzheimer’s disease [5], diabetes [6, 7] and metabolic disorders [8], glycosylation of proteins is changed dramatically. Analysis of glycosylation has the benefit that it provides information about the current status of the patient, which enables early diagnosis of disease onset and therapy monitoring.

Still major technological advances have to be made before glycoprotein biomarkers can be widely used in clinical diagnostics. In addition, clinical validation of potential biomarkers is equally important as well as the knowledge of how to interpret glycosylation abnormalities in the context of a certain disease. Important lessons can be learned by a comparison with a group of well-defined genetic defects, the Congenital Disorders of Glycosylation (CDG). CDG encompasses a group of inherited diseases with abnormal glycan metabolism. According to current nomenclature [9], they are divided into four different biochemical groups, comprising errors in protein N-linked glycosylation, protein O-linked glycosylation, glycolipid and GPI anchor glycosylation, and a group with defects in multiple glycosylation pathways [10]. Over 100 human genetic disorders have been associated with abnormal glycosylation [8, 11], of which at least 50 types are linked to deficient N-glycosylation of serum transferrin, historically referred to as CDG [12]. Because the defective genes are involved in a variety of functionally diverse metabolic pathways, the clinical presentation of CDG subtypes is highly heterogeneous. Clinical phenotypes range from mild to severe and involve multiple or only single affected organs. Examples of clinical symptoms include intellectual disability, seizures, muscle dystrophy, skeletal dysplasia, dysmorphic features, growth retardation, hematological and endocrine abnormalities, which show the widespread requirement of proper glycosylation for the human system [13, 14]. In line with these diverse clinical symptoms, diagnosis can be very challenging. Currently, only transferrin glycosylation analysis is widely used in clinical diagnostics for CDG.

Advanced mass spectrometry (MS) was introduced in CDG research to analyze released glycans from serum proteins [15–17] to gain more detailed insights in the glycan structural abnormalities. The growing contribution of mass spectrometry in the diagnosis of CDG is outlined in a recent review by Sturiale *et al.* [18]. Intact glycoprotein analysis of transferrin followed after the rapid development of mass spectrometry techniques and has now been added to routine diagnosis [19] and recently to therapy monitoring of CDG [20]. This method for protein-specific analysis has allowed high-resolution annotation of glycan structures, thereby revealing unique glycosylation profiles for specific genetic defects. This shows the potential to include mass spectrometry in specialized diagnostic laboratories and has already improved the diagnostic process by significantly shortening the time to reach a diagnosis.

It can be predicted that more glycosylation disorders will be reported in the near future since at least 2 % of the human genes encodes for proteins involved in glycan biosynthesis and recognition [8], and 5–10 % for proteins involved in Golgi homeostasis with a potential indirect effect on glycan metabolism. In addition, the wide-spread diagnostic application of next-generation sequencing is increasing the speed of CDG gene discovery [21]. The complexity of different glycosylation pathways, and the existence of tissue- and protein- specific glycosylation, indicates that the current use of transferrin as diagnostic marker is only a mere start to apply methods for glycoprotein analysis in a clinical diagnostic setting. In addition, the coming decade will show a merging of monogenic defects with genetic predisposition in more common diseases with abnormal protein glycosylation. Thus, there is a clear need to develop additional glycoprotein biomarkers to grasp the full scope of glycosylation defects in a diagnostic setting, and to learn how to interpret glycosylation abnormalities in the context of more common diseases.

Here, we will first describe the current state of diagnostics and gene identification for CDG. Secondly, we will discuss the potential use of existing plasma glycoprotein biomarkers, and finish with a look to the future in suitable mass spectrometry approaches for analysis of glycoproteins in clinical diagnostics.

## CDG diagnostics and gene identification

**Fig. 1:**
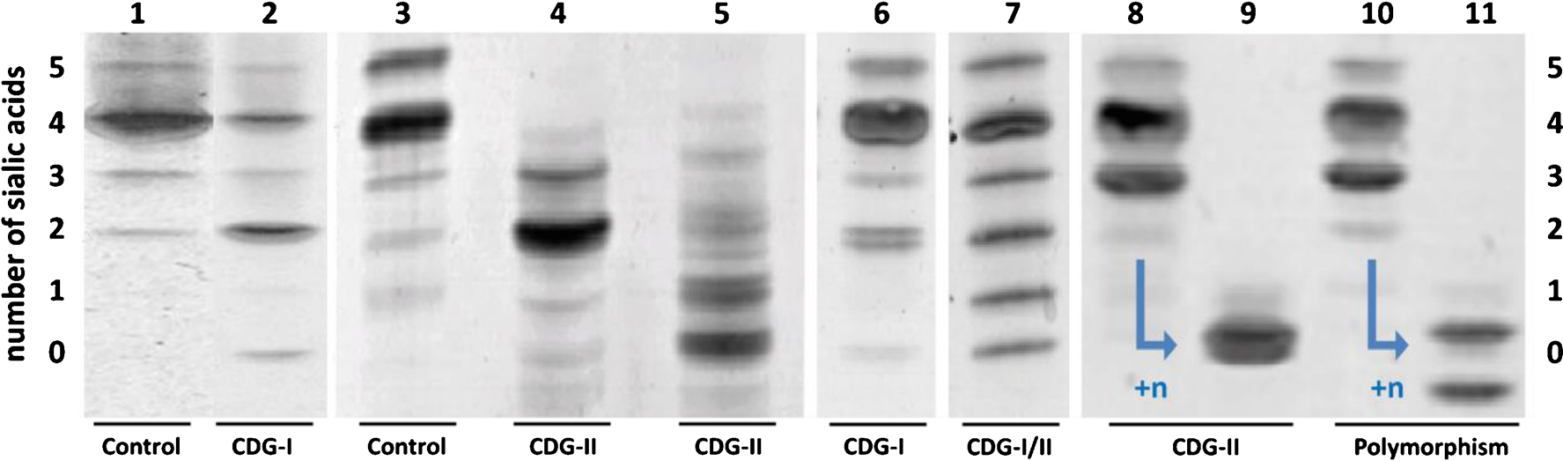
Serum transferrin isoelectric focusing (TIEF) gels with the number of terminal sialic acid residues indicated at the left and right side (0 to 5). Lanes 1 and 3: normal pattern; lane 2: CDG-I pattern (elevated asialo- and disialotransferrin bands); lanes 4, 5, 7 and 8: CDG-II pattern (additional increase of monosialo- and trisialotransferrin bands). Lane 6: mild CDG-I profile, resembles mild CDG-II pattern. Lane 7: combined CDG-I and -II profile. Lanes 8 to 11: transferrin before and after neuraminidase treatment (+n) for CDG-II defect MAN1B1-CDG and a polymorphism of transferrin, respectively. Two bands instead of one band will become visible when treating a transferrin polymorphism with neuraminidase

Defects in the N-glycosylation pathway are the best studied types of CDG. They are localized to the cytoplasm and the endoplasmic reticulum (ER) in CDG-I or to the cytoplasm and Golgi apparatus in CDG-II [22]. CDG-I defects are characterized by unoccupied glycosylation sites on proteins, thus lacking complete N-glycans, whereas CDG-II defects show immature, truncated glycans. Currently, the first line screening for these CDG subgroups in the majority of metabolic laboratories world-wide is based on the analysis of transferrin N-glycosylation by isoelectric focusing (TIEF), first introduced in 1984 by Jaeken *et al.* [23], by HPLC [24] or by capillary electrophoresis (CE) [25, 26]. These techniques allow separation of transferrin isoforms on basis of charge state determined by the number of terminal sialic acid residues (see Fig. 1). Normal plasma transferrin contains two complex type N-glycans with in total four terminal sialic acid residues as most dominant species (lanes 1 and 3). CDG patients show a decrease of this tetrasialo transferrin isoform and an increase of lower sialylated species. Abnormal profiles are classified as either CDG-I or CDG-II, depending on an isolated increase of asialo- and disialotransferrin bands (lane 2, CDG-I) or an additional increase of monosialo- and trisialotransferrin bands (lanes 4, 5 and 6, CDG-II), respectively [27]. Diagnostic interpretation of abnormal profiles can be complicated by the presence of transferrin polymorphisms that co-migrate with abnormally glycosylated transferrin isoforms [28, 29] (example in lane 8, Fig. 1). Treatment of serum transferrin (*in vitro*) with neuraminidase (sialidase), or alternatively analysis of parental plasma samples, is used to confirm or exclude a transferrin polymorphism [30] (lanes 6 to 9). Secondary causes of abnormal transferrin glycosylation are for example alcohol abuse, resulting in CDG-I profiles, the presence of sialidase in the plasma, or severe liver disease, both resulting in CDG-II profiles. In the majority of cases, CDG-I and -II profiles can be clearly discriminated. However, very mild CDG-I profiles can be interpreted as mild CDG-II (Fig. 1, lane 6) [31], and recently a first genetic defect was reported with a mixed CDG-I/II profile due to a combination of missing and truncated glycans (Fig. 1, lane 7) [20].

Conventionally, the follow up in case of CDG-I profiles consists of enzyme analysis in fibroblasts or leukocytes to diagnose PMM2-CDG, which is the most common type [32], or PMI-CDG. If excluded, the next step is analysis of dolichol- or lipid-linked oligosaccharides (LLO) in fibroblasts [33]. Nowadays, this is being replaced by whole exome sequencing using a filter for CDG-I genes [34], or targeted sequencing of a CDG-I gene panel. Alternatively, direct application of whole exome sequencing without prior CDG screening increasingly results in the identification of CDG defects, which need to be confirmed with Sanger sequencing. A combination of Capillary Electrophoresis [26] or LC [19] with mass spectrometry as a quick and sensitive test could functionally confirm the genetic defect. Also for confirmation of mild CDG-I profiles, mixed CDG-I/II profiles and even for further subtyping of CDG-I defects (such as ALG1-CDG, own data), high-resolution mass spectrometry of intact transferrin can be used.

The standard procedure for CDG-II patterns includes a follow up by isoelectric focusing of plasma apolipoprotein C-III (apoC-III-IEF) to discriminate between isolated N-glycosylation defects and a combined disorder of N- and mucin type O-glycosylation [35]. In addition, two-dimensional electrophoresis with immunoblotting was reported to separate and detect the isoforms of apoC-III, alpha-antitrypsin, alpha-1 acid glycoprotein, haptoglobin and transferrin [36]. Since CDG-II is characterized by truncated glycans, mass spectrometry methods can provide additional insights into the underlying genetic defect. Several methods have been reported for the analysis of transferrin glycopeptides [15, 37] and analysis of whole serum N-glycans [16, 38, 39] by MALDI-TOF mass spectrometry. With respect to O-glycans, mass spectrometry analysis of apoC-III [40] and combined mass spectrometry of released serum N- and O-glycans [41] have been reported. Recently, mass spectrometry analysis of apoC-III O-glycosylation was compared with two-dimensional electrophoresis by Yen-Nicolaÿ *et al.* and appeared to be a promising technique for analysis of defects in mucin O-glycosylation [42]. Application of high-resolution QTOF mass spectrometry for analysis of intact transferrin allowed to accurately annotate glycan structures on the level of the intact protein [31, 43] and proved highly efficient in subgrouping of CDG-II patients. For example, highly characteristic profiles can be observed for MAN1B1-CDG, PGM1-CDG with a characteristic combined CDG-I/II profile, and SLC35A2-CDG. The method has been validated for use in a clinical metabolic laboratory. The successful identification of gene defects directly from a characteristic glycoprofile of transferrin, has placed this method as second test immediately following CDG screening (diagnostic flowchart shown in [31]).

Currently, mass spectrometry of total serum N-glycans and of transferrin N-glycans has been included in only a few metabolic diagnostic laboratories. However, the high sensitivity and high specificity have already resulted in a significant shortening of the time to reach a genetic diagnosis in CDG, thereby showing the advantage to use mass spectrometry more broadly in CDG diagnostics.

## Therapy monitoring in CDG

Although many new glycosylation disorders have been identified in the last decade, only little progress has been made in development of new therapies. Different future therapeutic approaches have been proposed for the most common CDG type, PMM2-CDG, in a very comprehensive review by Freeze [44]. These include gene therapy, enzyme enhancement, enzyme replacement, bypassing the metabolic pathway and substrate supplementation. Most of these therapeutic strategies would be applicable to other CDG types as well, but are still under development. Several challenges have to be overcome, like the lack of appropriate model systems, high costs, small number of patients and the limited bioavailability of the drugs to reach their target, especially in regard to pass the blood–brain-barrier [44]. Only in four CDG subtypes, namely MPI-CDG, PGM1-CDG, SLC35C1-CDG and PIGM-CDG, a mechanism-based treatment has been applied [20, 45–48].

MPI-CDG is caused by a mutation in the cytosolic enzyme phosphomannose isomerase (MPI). The enzyme catalyzes the conversion of fructose-6-phosphate to mannose-6-phosphate [49], and therefore the disease is characterized by decreased mannose-6-phosphate. Mannose supplementation increased the mannose-6-phosphate levels and improved the clinical features in patients [45]. Despite mannose therapy, patients can develop progressive liver failure at a later stage of the disease. One first patient was successfully treated by liver transplantation, resulting in immediate recovery of most severe clinical symptoms [50]. As expected in view of the hepatic origin of transferrin, intact transferrin glycosylation profiles completely normalized after the transplantation. However, the disease status in other organs cannot be followed adequately by analysis of transferrin alone. This clearly shows the need for additional biomarkers for CDG therapy monitoring, including glycoproteins that are synthesized by other tissues and cell types.

**Fig. 2:**
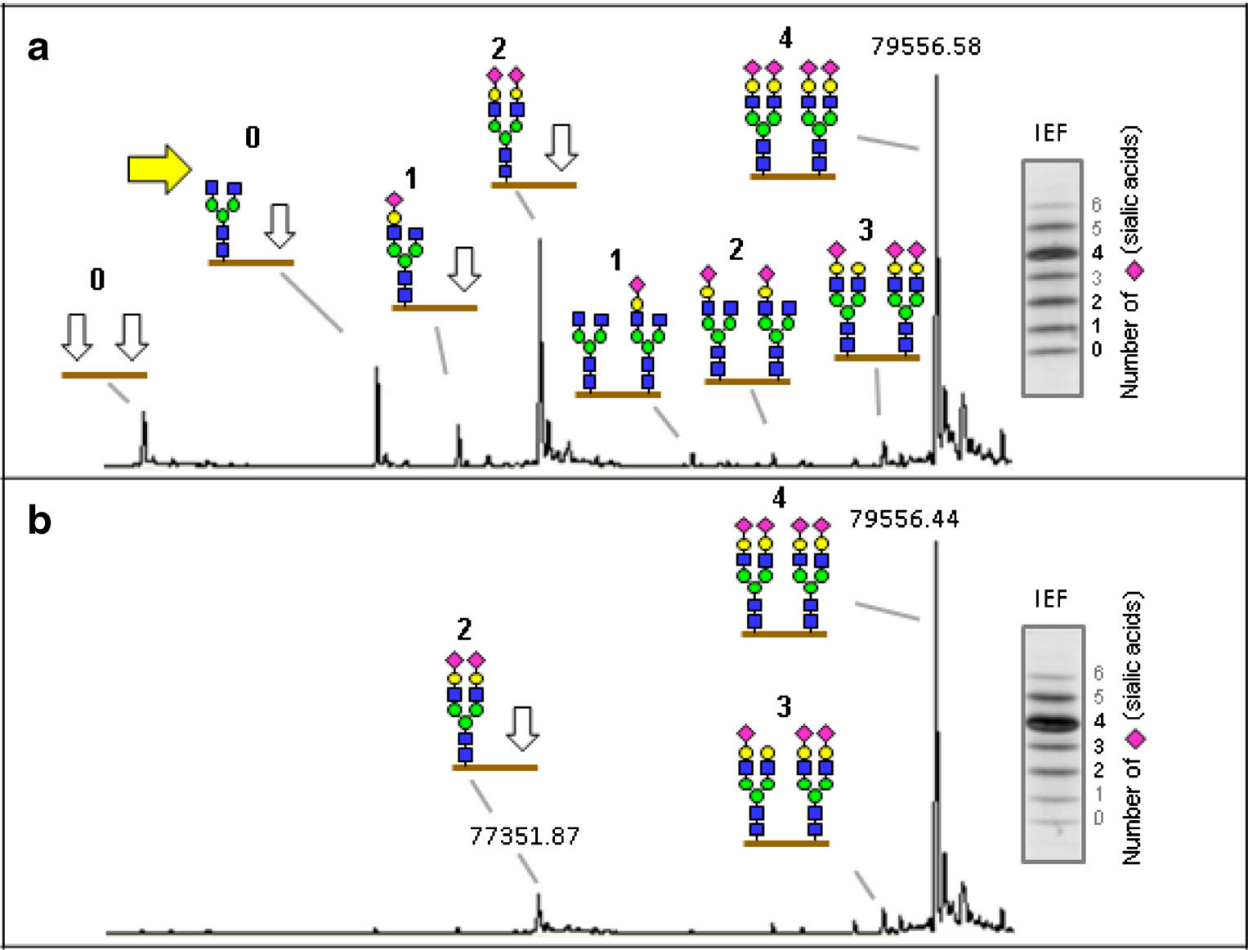
Effects of Dietary Galactose on Glycosylation. High resolution mass spectrometry showing glycan structures of transferrin before (**a**) and after (**b**) 2 weeks intake of supplementary galactose and corresponding patterns of transferrin isoelectric focusing (IEF). The protein backbone is symbolized by a brown horizontal line. The unoccupied positions are indicated by open arrows () and define the CDG-I-component of this phenotype. The yellow arrow () demonstrates the absence of galactose on one of the truncated glycans, which define the CDG type-II component. The number of sialic acids is indicated above each structure, and the insets at the right show respective IEF results

PGM1-CDG is caused by a mutation in the phosphoglucomutase 1 enzyme (PGM1). The enzyme catalyzes the interconversion of glucose-6-phosphate and glucose-1-phosphate [51]. The disease was identified by a highly characteristic transferrin glycosylation profile with high-resolution mass spectrometry, which showed a combination of CDG-I and CDG-II. The spectra showed truncated glycans lacking galactose. Based on the spectra and known metabolic pathways, supplementation of patients with galactose resulted in almost complete normalization of transferrin glycosylation [20] (Fig. 2). The normalization of the truncated glycans upon galactose treatment can be explained based on known metabolic pathways. However, the normalization of the complete glycans (CDG-I) cannot be fully explained. More detailed insight into the dynamics and regulation of sugar metabolism, and its link with protein glycosylation, would help us to better understand the glycosylation machinery, further optimize therapy and develop new therapies for other CDG patients. In addition, monitoring of the glycosylation of additional proteins is required to learn if other tissues apart from liver respond equally well to galactose treatment.

SLC35C1-CDG is caused by a mutation in the GDP-fucose transporter in the Golgi-membrane, resulting in hypofucosylation of N-glycopeptides [52]. Patients typically lack sialyl-Lewis X (sLex) structures on the cell surface of neutrophils, which is required for their rolling on endothelial cells before extravasation [53]. This CDG type is therefore also known as leukocyte adhesion deficiency II (LAD II) and leads to infectious complications. Depending on the nature of the mutation, some patients respond to L-fucose application [46, 54]. However, no proper glycoprotein marker is currently available to monitor therapy response and disease progression.

PIGM-CDG is a defect in GPI-anchor biosynthesis and is caused by a mutation in the glycosylphosphatidylinositol glycan (class M) (PIGM) promoter. PIGM transfers the first mannose to the GPI anchor at the luminal side of the endoplasmic reticulum [55]. Mutation in the promoter causes histone hypoacetylation, resulting in defective GPI anchored proteins on blood cells [47]. A patient was therefore treated with sodium phenylbutyrate, which is a histone deacetylase inhibitor. This increased PIGM transcription, resulting in improved GPI anchored glycoproteins: CD59 (MAC-inhibitory protein) on erythrocytes and CD59 and CD24 (heat stable antigen) on granulocytes (FACS analysis) [55]. Clinically, the patient improved and was able to walk again and had no more seizures [48].

Currently, CDG therapy monitoring is mainly based on clinical features, transferrin analysis or flow cytometry of several glycoprotein surface markers on blood cells. The main advantage of using transferrin as a biomarker for CDG is its high abundance in blood, allowing fast, detailed and sensitive detection of N-glycosylation in small sample volumes. Also for treatment of fructosemia, a common secondary cause of CDG, the loss of intact glycans can be monitored sensitively by high resolution mass spectrometry of transferrin [31]. However, therapy monitoring in clinical diagnostics is very limited with the analysis of transferrin, and additional biomarkers are essential for the development and monitoring of new and current therapies.

## Novel glycoprotein biomarkers for CDG

### The need for glycoprotein biomarkers beyond transferrin

Although transferrin is a very useful diagnostic biomarker for most CDG types with deficient N-glycosylation, several defects in the N-glycosylation pathway are known with a normal transferrin profile. These include both CDG-I subtypes (TUSC3-CDG, ALG13-CDG, ALG14-CDG), Golgi glycosylation defects (GCS1-CDG, SLC35A3-CDG and SLC35C1-CDG), as well as defects in sugar metabolism (GNE, PGM3 and GFPT1). The growing list of these type of defects with normal transferrin glycosylation indicates that additional proteins have to be included for correct diagnosis. Since glycans can be expressed in a tissue-specific way [56–58], defects in their synthesis will be missed when analyzing only hepatocyte-derived transferrin. Furthermore, analysis of defects in the synthesis of glycan structures not or hardly present on transferrin (such as fucose in SLC35C1-CDG), will require inclusion of additional proteins. Additionally, recent studies indicate the existence of regulatory processes in protein-specific glycosylation that we don’t yet understand. For example, apart from the normal glycosylation of transferrin, glycosylation of IgG was abnormal in GCS1-CDG patients [59], although GCS1 is ubiquitously expressed. Cohen syndrome due to mutations in *VPS13B* shows normal transferrin glycosylation as well, however, Duplomb *et al.* found abnormal N-glycan structures by mass spectrometry of total serum glycans [60]. They identified a decrease in sialylation and galactosylation of total serum N-glycans, which indicates deficient Golgi N-glycosylation of specific proteins. They propose to analyze the highly glycosylated protein intercellular cell adhesion molecule 1 (ICAM-1) and lysosome-associated membrane protein 2 (LAMP-2) in blood cells as a pre-screening test for Cohen syndrome.

In addition to defects in the N-glycosylation pathway, there are many other genetic glycosylation defects (*e.g*., in O- and lipid-glycosylation, and synthesis of glycosaminoglycans and glycophosphatidylinositol (GPI) anchors) for which specific markers need to be developed, which would be of similar value as transferrin for defects in the N-glycosylation pathway. In conclusion, there is a clear need for additional glycoprotein biomarkers, in addition to transferrin, for efficient diagnostics of the full scope of genetic glycosylation defects.

### Potential glycoprotein biomarkers for diagnostic application in CDG

An extensive number of glycoprotein biomarkers is already described in the literature for diagnosis of other types of diseases, particularly for diabetes mellitus [61], Alzheimer’s disease [62], rheumatoid arthritis or other inflammatory diseases [63, 64], and cancer [65]. These proteins could be a potential glyco-marker for CDG as well, because they are readily measurable in biological matrices, and they have different type of glycosylation, or are from other origin compared to transferrin.

The analysis of glycoprotein levels of some cancer biomarkers is FDA approved as prognostic or diagnostic screening [66–68], indicated by an asterisk in the table below. The detection of most glycoproteins is still mainly based on immunoassays and electrophoresis techniques, although the application of mass spectrometric screening is starting to be explored as well. To highlight the extent of today’s glycoproteomic biomarker research, Table 1 provides an overview of some key examples of candidate glycoprotein biomarkers from recent literature for the previously mentioned diseases.

**Table 1:** Overview of some key examples of serum glycoprotein biomarkers from recent literature for diseases that present with altered glycoprotein levels and glycosylation abnormalities

Glycoprotein Biomarker	Disease	Detection on protein levels or altered glycoforms	Detection technique	Reference
Transferrin^a^	– CDG	– Glycosylation abnormalities	– IEF, QTOF	[50]
	– Sepsis	– Decrease in sialylation	– ELLA	[93]
	– Acute pancreatitis	– Increased fucosylation	– LC-MS/MS	[94]
	– Ovarian cancer	– Increase of concentration	– ELISA	[95]^a^
α1-antitrypsin	– CDG	– Altered glycosylation	– 2DE	[36]
	– Chronic obstructive pulmonary disease (COPD)	– Decreased concentration	– ELISA	[96]
	– Liver cirrhosis and liver cancer	– Core and outer arm fucosylation	– 2DE, MALDI-TOF – FLISA	[97]
	– Lung cancer	– Altered glycosylation	– Lectin microarray, ELISA	[98]
Haptoglobin	– CDG	– Altered glycosylation	– 2DE	[36]
	– Long cancer	– sLe^X^ increase	– HILIC, WAX-HPLC	[99]
	– Acute phase ovarian cancer	– sLe^X^ increase	– 2DE, NP-HPLC	[100]
	– Stomach cancer	– sLe^X^ increase	– 2DE, LC-MS/MS – ELISA – HILIC	[101]
	– Pancreatic cancer	– Fucosylation	– MALDI-TOF – Immunoblot	[102, 103]
	– Rheumatoid arthritis	– Reduced mannosylation	– 2DE, MALDI-TOF – WAX-HPLC – ELISA	[104]
	– Pancreatic cancer	– Changes in sialylation	– LC-MS/MS	[94]
	– Gastric cancer	– Increase of concentration	– ELISA – MRM	[105]
Fetuin	– Hepatocellular carcinoma (HCC)	– Fucosylation	– LC-MS/MS – FLISA	[106]
	– Chronic pancreatitis, pancreatic cancer	– sLe^X^ increase	– 2DE, WAX-HPLC, NP-HPLC, LC-MS/MS, MALDI-TOF	[107]
α-1-acid glycoprotein	– CDG	– Altered glycosylation	– 2DE	[36]
	– Acute phase ovarian cancer	– sLe^X^ increase	– 2DE, NP-HPLC	[100]
	– Chronic pancreatitis, pancreatic cancer	– Increased branching and sLe^X^	– 2DE, WAX-HPLC, NP-HPLC, LC-MS/MS, MALDI-TOF	[107]
	– Rheumatoid arthritis	– Altered mannosylation	– 2DE, MALDI-TOF – WAX-HPLC – ELISA	[104]
Immunoglobulin G (IgG)	– Acute phase ovarian cancer	– sLe^X^ increase	– 2DE, NP-HPLC	[100]
	– Advanced ovarian cancer	– Reduced galactosylation and sialylation levels	– 2DE, NP-HPLC	[100]
	– Stomach cancer	– Core fucosylation	– 2DE, LC-MS/MS	[101]
α1-antichymotrypsin	– Acute phase ovarian cancer	– sLe^X^ increase	– 2DE, NP-HPLC	[100]
	– Alzheimer’s disease	– Concentration	– Immunoassay	[108]
α_2_-macroglobulin	– Sjögren’s syndrome	Abnormal glycosylation	Immunoblot	[109]
α-fetoprotein^a^	– Liver diseases	– Core fucosylation, concentration	– 2DE, immunoblot	[110]
	– Liver cancer^a^	– Concentration	– Anti-body (liquid phase binding assay)	[67]^a^
des-γ-carboxypro-thrombin	Liver diseases, liver cancer	Core fucosylation	– 2DE, immunoblot	[110]
Ferritin	Still’s disease, hemophagocytic syndrome	Altered glycosylation	Immunoassay	[111]
Ceruloplasmin	– CDG	– Low levels, glycosylation abnormalities	– 2DE	[50]
	– Hepatocellular carcinoma (HCC)	– Upregulated, core fucosylation	– LC-MS/MS – 2DE, MALDI-TOF	[112, 113]
	– Pancreatic cancer	– sLe^X^ increase	– LC-MS/MS – Immunoblot	[114]
Thyroglobulin^a^	– CDG	– Low levels, glycosylation abnormalities	– 2DE	[50]
	– Thyroid cancer	– Increase of concentration	– Immunoassay	[115], [68]^a^
Thyrotropin, Thyroid stimulating hormone	– CDG	– Low levels, altered glycosylation	– IEF, serum levels	[50, 116, 117]
	– Thyroid function	– Increase of glycoprotein concentration	– Immunoassay	[118]
Hemopexin	Hepatocellular carcinoma (HCC)	Fucosylation	– LC-MS/MS – FLISA	[106]
Clusterin	– Stomach cancer	– Smaller N-glycans	– 2DE, LC-MS/MS	[101]
	– Gastric cancer	– Decrease of concentration	– ELISA – MRM	[105]
	– Clear cell renal cell carcinoma	– Decreased (core fucosylated) biantannary glycans	– SDS-PAGE, LC-MS/MS – Immunoblot	[119]
Leucine rich-α2-glycoprotein	Stomach cancer	Upregulation, altered glycosylation	2DE, LC-MS/MS	[101, 120]
α-dystroglycan	Walker-Warburg syndrome	Hypoglycosylation	Immunoassay	[121]
Kininogen	– Colorectal cancer (CRC)	– Elevated sialylation and fucosylation	– LC-MS/MS – Lectin microarray	[122]
	– Colorectal cancer (CRC)	– Increase of concentration	– MALDI-TOF – ELISA – Immunostaining	[123]
Kallistatin	Liver cirrhosis	Increase of concentration	ELISA	[124]
Afamin	– Metabolic syndrome	– Increase of concentration	– ELISA	[125]
	– Gastric cancer	– Decrease of concentration	– ELISA – MRM	[105]
Prostate Specific Antigen (PSA)^a^	Prostate cancer	Increase of concentration	Immunoassay	[126], [68]^a^
Human chorionic gonadotrophin (hCG)	– Ovarian tumors – Testicular tumors	Increase of concentration	Immunoassay	[68], [66]^a^
Apolipoprotein (A-1)^a^	Ovarian cancer	Decrease of concentration	ELISA	[95]^a^
Transthyretin, prealbumin^a^	Ovarian cancer	Decrease of concentration	ELISA	[95]^a^
β2-microglobulin^a^	Ovarian cancer	Increase of concentration	ELISA	[95]^a^
Cancer antigen 125 (CA125) or MUC16^a^	Ovarian cancer	Increase of concentration	ELISA	[67, 95]^a^
Carbohydrate antigen 19–9 (CA19-9)^a^	– Pancreatic cancer Ovarian cancer	sLe^a^ on mucin glycoproteins	ELISA	[64, 68]^a^
Cancer antigen 15–3 (CA15-3)^a^	Breast cancer	Sialylated O-linked oligosaccharide on MUC1	ELISA	[67, 68]^a^
CA27-29^a^ (MUC1)	Breast cancer	Protein concentration	ELISA	[67, 68]^a^

*2DE* 2D-electrophoresis; *ELLA* enzyme-linked lectin assay; *FLISA* Lectin-Fluorophore-linked Immunosorbent Assay; *HILIC* Hydrophilic Interaction Liquid Chromatography; *sLe* ^*a*^ Sialyl Lewis A structures; *sLe* ^*X*^ Sialyl Lewis X structures; *WAX-HPLC* weak anion exchange high performance liquid chromatography

^a^FDA approved

As can be seen from this overview, the number of glycoproteins as diagnostic biomarker is growing for multiple diseases, see Table 1 for details and references. For some of these glycoproteins, altered glycosylation has already been investigated in more detail using mass spectrometry. In particular core or sLe^X^ fucosylation seems to be a frequent significant change observed. It should also be noted that the glycoprotein level is often the diagnostic parameter. So ideally, both protein levels and glycosylation analysis should be included in one assay. It could be envisioned that combination of several plasma proteins with a more heterogeneous glycan composition and increased fucosylation as compared to transferrin, can be used in a panel to further increase the diagnostic efficiency for CDG-I and CDG-II. Apart from selections based on biological aspects as discussed in section 4.1, an additional challenge is the mass spectrometric compatibility and the different approaches for analysis, which will be discussed in the next paragraph.

## Mass spectrometry in glycoprotein analysis

**Fig. 3:**
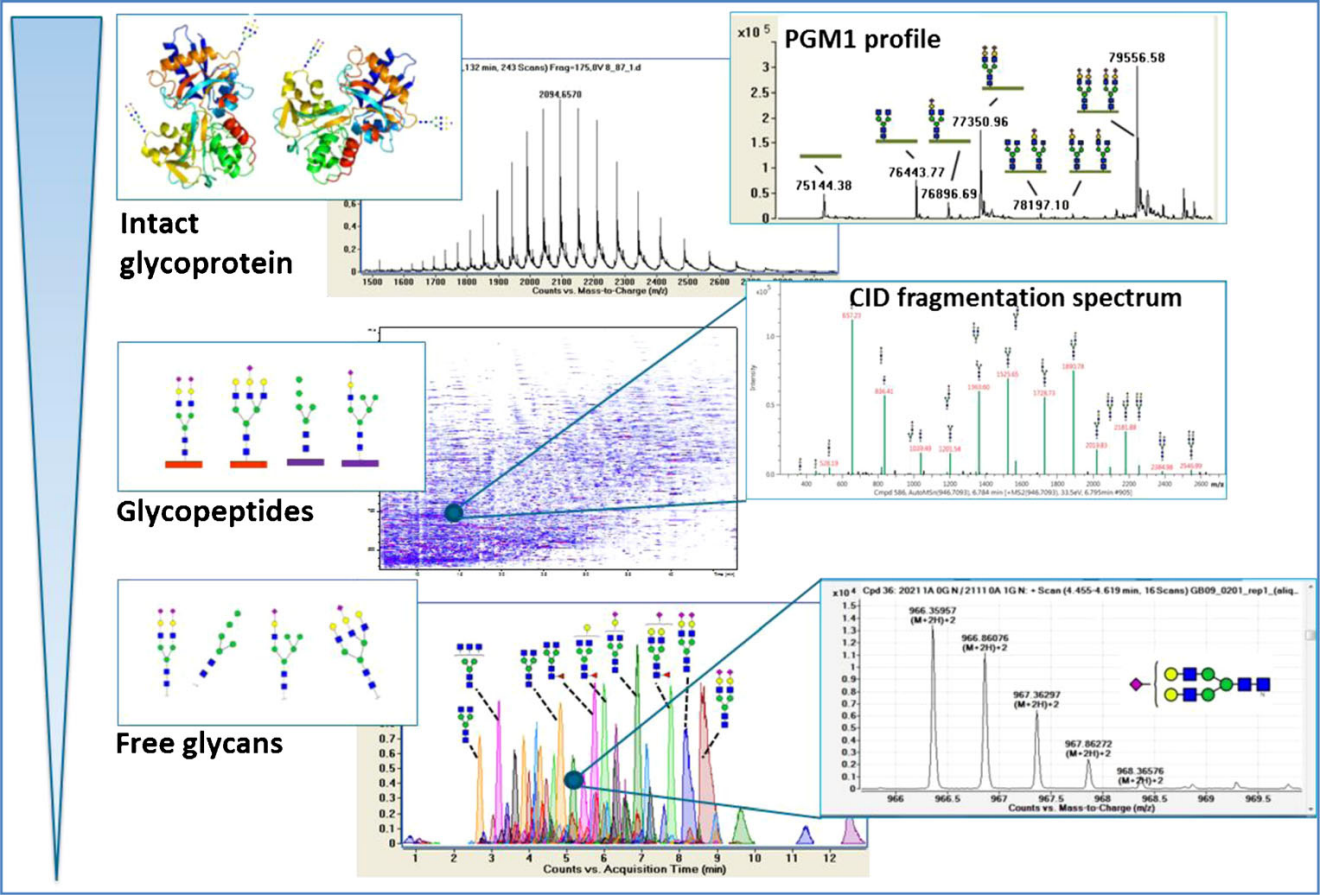
Schematic representation of the different types of glycoprotein analysis using mass spectrometry

A growing number of protein biomarkers are analyzed in the clinics by mass spectrometry. Mass spectrometry is ideally suited for the clinical laboratory, because the technique is sensitive, compatible with biological matrices, suitable for high-throughput analyses and it provides detailed structural information. The technique has proven itself for CDG diagnostics through the routine analysis of serum transferrin, which will pave the way in the search for additional glycoprotein biomarkers. In Fig. 3, an overview is depicted of the different levels of glycosylation analysis by mass spectrometry, better known as Glycomics. Intact glycoprotein analysis provides quantitative glycan structural information of the intact glycoprotein. Quantification is possible with the assumption that ionization of the molecules dominated by the amino acid backbone. Loss of complete glycans can therefore accurately be detected. Intact protein analysis is limited by size and hydrophobicity of the protein, and limited by the number of glycosylation sites for correct interpretation of the spectra. Glycopeptide analysis provides detailed site-specific information on glycan forms, and enzymatic digestions increase the chance to measure large or hydrophobic proteins of interest. However, the ionization efficiency is influenced by the type of glycan, which hampers any quantitative comparison between different glycoforms. Analysis of free glycans allows sensitive detection of very low abundant glycan structures, however information on the corresponding protein/peptide attachment site is lost. Therefore, each detection technique has its own strengths and weaknesses depending on the intended goal of the analysis. Below, we discuss the use of mass spectrometry in biomarker discovery and for its potential use as a diagnostic test.

### Glycoprotein biomarker discovery

The most widely used methods for biomarker discovery within the field of glycomics includes free N-glycan profiling of a biological fluid, such as serum, plasma or cerebrospinal fluid. Many groups have published on the discovery of altered glycosylation in a wide variety of diseases, and convincingly showed that glycans have potential as biomarkers, being highly sensitive to pathological changes [69–73]. Thus far, only a very limited number of potential biomarkers based on free glycan profiling is further developed and used in a real clinical diagnostic test, one example being the GlycoCirrhoTest [74] for distinguishing cirrhotic from non-cirrhotic chronic liver disease patients.

Two major limitations of total serum N-glycan analysis create the basis for improvement: First, the information to which proteins glycans were attached, is lost in total serum N-glycan analysis. Secondly, alterations in glycosylation are biased by fluctuations in the dynamic N-glycoproteome, which are dependent on environmental factors such as an immune response [75–78]. Ideally, profiling methods need to be used that take into account the protein to which the altered glycans are attached, such as profiling of intact glycopeptides from complex biological samples. Recent technical developments and improved databases seem to make this challenge feasible. A combination of collision induced dissociation (CID) and electron-transfer dissociation (ETD) fragmentation techniques is preferred to characterize glycopeptides [79, 80]. While CID provides information related to the composition and structure of glycan moieties attached to a peptide backbone, ETD fragments mainly the peptide backbone and leaves the glycan intact, permitting *de novo* sequencing of the peptide moiety [81, 82]. Due to the relative low abundance and low ionization efficiency of glycopeptides, glycoproteins need to be enriched, digested and chromatographically separated for this type of analyses. Since the glycan moiety makes the glycopeptide generally more polar, HILIC is a commonly used enrichment strategy for glycopeptides [83]. Other usual approaches are immune-affinity (reactive beads) [84, 85] or lectin-affinity purifications [86, 87]. Furthermore, commercial kits are available to deplete serum samples from the most abundant serum proteins, although that might cause potential biomarkers (see Table 1) to be lost. Combining these techniques enables unambiguous in-depth identification of both the peptide and the glycan. However, the data analysis software is not yet sophisticated enough to enable automated sequencing of peptides and combining search results from both fragmentation techniques for identification. Therefore, intact glycopeptide analysis from complex biological samples is still far from being a routine diagnostic analysis. Within a research environment, glycopeptide profiling is highly promising for the identification of new glycoprotein biomarkers.

One first example of glycopeptide analysis using the combination of CID and ETD fragmentation, is a very recent study of Medzihradszky *et al.* on tissue-specific glycosylation [58, 88]. They compared the glycosylation patterns of mouse liver with brain tissue and not only found that these patterns differ by the cellular location of the glycoprotein (*e.g*., ER, lysosomal or transmembrane), but also showed tissue-specific differences for the same protein.

### Glycoprotein biomarker application in clinical diagnostics

Diagnostic assays are required to be fast, robust and suitable for high-throughput measurements, while in addition the interpretation in a clinical setting should be straightforward. Free N-glycan analysis meets the technical requirements, however, diagnostic interpretation is highly complicated due to the many causes that influence N-glycan profiles. For some well-defined CDG subtypes with a clear genetic defect, such as B4GALT1-CDG and MGAT2-CDG, interpretation is clear. However, abnormalities in free N-glycan profiles for other defects are more subtle or overlapping with multiple defects. Examples include PGM1-CDG, where the lack of complete glycans is not found in total serum N-glycan profiles and only minimal loss of galactose is not sufficiently specific to reach a diagnosis.

Diagnostic application of glycopeptides is of particular interest, because multiple identified biomarkers could subsequently be used for diagnostic high-throughput screening by means of multiple/serial reaction monitoring (MRM/SRM), similar as is increasingly used for the detection of protein biomarkers in clinical diagnostics. In this sensitive targeted analysis, specific (transitions of) predefined precursor ions are sequentially selected for MS/MS detection in a quantitative and reproducible manner [89]. For instance, Hong *et al.* developed an MRM analysis to analyze the IgG free glycans in whole serum. By normalizing protein glycosylation to absolute protein concentrations they were able to examine quantitative changes in glycosylation at site-specific level [90], which is crucial to distinguish between altered glycosylation and change in protein levels. Hulsmeier *et al.* published a MRM method to quantify the N-glycosylation site occupancy for transferrin and α_1_-antitrypsin, and found out that the site occupancy correlated well with the severity of the disease [91]. Moreover, they observed preferences for specific occupation sites. The method could in principle easily be extended to multiple glycoproteins.

Very recently, Sun *et al.* proposed a new method to measure absolute protein glycosylation stoichiometry in complex biological samples [92]. They use the relative ratios between the N-glycosylated peptide, the peptide without the glycans and the complete glycoprotein from the same type of biological sample to profile the glycosylation occupancy and glycoform stoichiometry and determined 117 absolute N-glycosylation occupancies in OVCAR-3 cells. Application to CDG has not been tested yet, but might be promising in biomarker discovery.

A specific set of intact glycoproteins would have several advantages over glycopeptide mixtures, mainly improving on the relative quantification. One needs to consider that transferrin is a relative simple glycoprotein to analyze as it has only two glycosylation sites, whereas other proteins might go up to multiple glycosylation sites and increased glycan heterogeneity. Each additional glycosylation site will complicate annotation [83]. In addition, an antibody would be required to affinity-purify the protein from the biological complex sample. The antibody would need to have its epitope outside of any glycosylation motif, to ensure glycosylation-independent purification of all glycoforms.

Overall, each method has its strengths and weaknesses. Targeted, MRM based mass spectrometry of glycopeptides is fast, robust and sensitive, allowing complex mixtures to be analyzed. With intact glycoprotein analysis, a limited number of proteins can be analyzed. However, all glycoforms of one protein are measured simultaneously in the mass spectrometer, which allows relative quantification of glycoforms and easy comparison of both CDG type-I and CDG type-II defects in one spectrum.

## Outlook

The ultimate goal in clinical diagnostics of CDG would be to cover the complete field of glycosylation disorders in a single or only few assays. This would not only benefit diagnostics of monogenic, inherited disorders of glycosylation, but also the more common, complex genetic disorders like cancer, Alzheimer or inflammatory diseases. Several diseases show abnormal glycosylation, and an increasing number of glycosylation disorders is identified by next generation sequencing techniques. More and more defects within the newly discovered disorders are found outside the pathway of protein N-glycosylation, for example defects in energy metabolism, or GPI-defects. To include these defects, the repertoire of glycoprotein biomarkers should be expanded, by analyzing a well-defined set of multiple, intact biomarker glycoproteins.

Table 1 provides an overview of glycoprotein biomarkers, which are worth considering for clinical diagnostics of CDG. It lists candidates which are already applied in clinical laboratories for other types of diseases, so addition of these proteins should be feasible. Our preference is to include several proteins in one assay, either as glycopeptides or as intact proteins. The intact transferrin spectra already show the potential to develop a unique classification system for CDG. The spectra provide such a characteristic profile that clinical diagnostics is possible at a glance [20, 43].

When glycoproteins are digested, housekeeping proteins or non-glycosylated peptides of a targeted glycoprotein are necessary for normalization and quantification purposes. In addition, improved ionization and enhanced separation for low abundant glycopeptides might be required. When followed by high-resolution detection and adequate databases for automated searches, automated MRM analyses can be used as a fast diagnostic test. Until then, we propose that intact glycoprotein profiling of well-defined protein mixtures will have the best potential for CDG diagnostics and therapy monitoring in the near future.

In conclusion, simultaneous analysis of multiple glycoproteins would highly increase our knowledge of the glycosylation machinery in different types of cells, and improve our diagnostic power to sensitively and specifically monitor disease in CDG patients. A highly detailed glycosylation read-out of individual proteins would work in synergy with Next Generation Sequencing techniques in the identification of new inherited disorders. Also, the diagnostics of more common disorders shall benefit from the lessons that will be learned from the analysis of complex glycoprotein mixtures.

## Abbreviations

2DE: 2D-electrophoresis
apoC-III: apolipoprotein CIII
CA125: cancer antigen 125
CA15-3: cancer antigen 15–3
CA19-9: carbohydrate antigen 19–9
CA27-29: cancer antigen 27–29
CD24: cluster of differentiation 24 or heat stable antigen CD24 (HSA)
CD59: MAC-inhibitory protein (MAC-IP)
cdg: Congenital Disorders of Glycosylation
CDG-I: Congenital Disorders of Glycosylation type I
CDG-II: Congenital Disorders of Glycosylation type II
CID: collision induced energy
COPD: Chronic obstructive pulmonary disease
CRC: Colorectal cancer
ELISA: Enzyme Linked Immunosorbent Assay
ELLA: enzyme-linked lectin assay
ETD: electron transfer dissociation
FACS: fluorescence activated cell sorting
FDA: US Food and Drug Administration
FLISA: Lectin-Fluorophore-linked Immunosorbent Assay
FRU: fructose
FUC: fucose
GAL: galactose
GDP: guanosine diphosphate
GFPT1: Glutamine-Fructose-6-Phosphate Transaminase 1
GLC: glucose
GNE: UDP-N-acetylglucosamine 2-epimerase/N-acetylmannosamine kinase
gpi: glycosylphosphatidylinositol
HCC: Hepatocellular carcinoma
hCG: Human chorionic gonadotrophin
HILIC: Hydrophilic Interaction LIquid Chromatography
ICAM-1: intercellular cell adhesion molecule 1
IEF: Iso Electric Focusing
IgG: Immunoglobulin G
LAD-II: leukocyte adhesion deficiency II
LAMP-2: lysosome-associated membrane protein 2
LC: Liquid Chromatography
LC-MS: Liquid Chromatography - mass spectrometry
LLO: Lipid linked oligosaccharide
MAC: membrane attack complex
MALDI-TOF: matrix assisted laser desorption ionisation - Time-of-Flight
MAN: Mannose
MRM: multiple reaction monitoring
MUC16: mucin 16 or ovarian cancer-related tumor marker CA125
NP-HPLC: normal phase - high pressure liquid chromatography
PSA: prostate specific antigen
QTOF: quadrupole time of flight
SRM: single reaction monitoring
sLe^X^: Sialyl Lewis X structures
sLe^a^: Sialyl Lewis A structures
tief: transferrin isoelectric focusing
UDP: uridine diphosphate
UPLC-MS: ultra high pressure liquid chromatography- mass spectrometry
WAX-HPLC: weak anion exchange high performance liquid chromatography
ᅟ: **List of human genes**
ALG13: subunit of UDP-*N*-acetylglucosamine transferase complex
ALG14: subunit of UDP-*N*-acetylglucosamine transferase complex
ALG1: mannosyltransferase 1
ALG8: glucosyltransferase 2
DOLK: dolichol kinase
GCS1: mannosyl-oligosaccharide glucosidase
MAN1B1: alpha-1,2-mannosidase
MPI: mannose phosphate isomerase
PGM1: phosphoglucomutase 1
PGM3: phosphoglucomutase 3
PIGM: glycosylphosphatidylinositol
PMI: Phosphomannose isomerase
PMM2: phosphomannomutase
SLC35A2: UDP-galactose transporter
SLC35A3: UDP-*N*-acetylglucosamine transporter
SLC35C1: GDP-fucose transmembrane transporter
TUSC3: Oligosaccharyltransferase TUSC3 subunit
VPS13B: Vacuolar Protein Sorting 13 Homolog B
